# Pathological and Structural Alterations of the Visual Pathway in APP/PS1 Mice: A Spatiotemporal Analysis

**DOI:** 10.3390/diagnostics15212768

**Published:** 2025-10-31

**Authors:** Jingan Chen, Yang Xia, Ke Chen, Dezhong Yao

**Affiliations:** 1 Institute of Basic Medical Sciences, Chinese Academy of Medical Sciences, School of Basic Medicine Peking Union Medical College, Beijing 100005, China; b2021005040@student.pumc.edu.cn; 2 Research Unit of NeuroInformation (2019RU035), Chinese Academy of Medical Sciences, University of Electronic Science and Technology of China, Chengdu 611731, China; xiayang@uestc.edu.cn; 3 MOE Key Lab for NeuroInformation, School of Life Science and Technology, University of Electronic Science and Technology of China, Chengdu 611731, China; 4 Sichuan Provincial Key Laboratory for Human Disease Gene Study and the Center for Medical Genetics, Sichuan Academy of Medical Sciences, Medical School, University of Electronic Science and Technology of China, Chengdu 610072, China

**Keywords:** Alzheimer’s disease, visual pathway, primary visual cortex, behavioral assessments, histological staining, pathology, structure, APP/PS1 mice

## Abstract

**Background/Objectives**: Visual dysfunction emerges during the mild cognitive impairment stage of early Alzheimer’s disease (AD). While previous studies have primarily focused on retinal pathology, the early pathological progression across central nodes of the visual pathway remains inadequately characterized. This study examined regional pathological and structural alterations throughout the visual pathway at different disease stages in APP/PS1 transgenic mice aged 3, 6, and 9 months. **Methods**: Cognitive function was first assessed using novel object recognition and Y-maze tests to stage disease progression. Subsequently, Histological staining was employed to systematically analyze pathological features in the retina, lateral geniculate nucleus (LGN), and primary visual cortex (V1). Evaluated parameters encompassed β-amyloid (Aβ) deposition levels, microglial activation status, total neuronal counts, parvalbumin (PV)-positive neuron numbers, and tissue thickness measurements of the retina and V1. **Results**: At 6 months, mice exhibited an early symptomatic phenotype with selective spatial working memory deficits while long-term memory remained intact. Pathological analysis revealed concurrent Aβ deposition and microglial activation in V1, retina, and hippocampus by 6 months, whereas comparable LGN changes manifested only at 9 months, demonstrating regional heterogeneity in disease progression. V1 neuronal populations remained stable through 6 months but showed significant reduction by 9 months, though PV-positive neurons were selectively preserved. The LGN exhibited no neuronal loss even at 9 months. Gross structural thickness of both retina and V1 remained unchanged across all timepoints. **Conclusions**: These findings demonstrate that early visual system pathology in this AD model extends beyond the retina. The primary visual cortex exhibits early pathological changes (Aβ deposition and neuroinflammation) concurrent with hippocampal involvement, progressing to selective neuronal loss in later stages. The severity and selectivity of V1 pathology surpass those observed in other visual pathway nodes, including the LGN. Thus, V1 could represent not merely an affected region but a promising site for elucidating early cortical AD mechanisms and developing novel diagnostic biomarkers.

## 1. Introduction

Alzheimer’s disease (AD) is the most common neurodegenerative disorder worldwide, and as of 2019, more than fifty million people were affected by dementia globally; this number is projected to triple by 2050 [1]. Although the hallmark symptoms of AD are cognitive decline and memory impairment, increasing evidence indicates that sensory deficits, particularly visual dysfunction, emerge several years before the onset of cognitive symptoms [2]. These visual impairments include deficits in contrast sensitivity [2,3,4], color perception [2,3], visuospatial function [5], and visual field loss [6], all of which severely compromise patients’ quality of life. Such early visual abnormalities in AD (i.e., during mild cognitive impairment) are receiving growing attention and are being proposed as emerging early biomarkers [6,7,8,9,10].

The retina is regarded as a specialized extension of the central nervous system and shares the same embryological origin as the brain; therefore, it has attracted considerable attention as a non-invasive window for detecting AD pathology [11]. Previous studies have documented significant pathological changes in the retina of AD patients, including loss of retinal ganglion cells, thinning of the retinal nerve fiber layer, accumulation of retinal Aβ, and microvascular abnormalities [11,12]. Numerous reports indicate that retinal ganglion cells (RGCs), especially intrinsically photosensitive retinal ganglion cells (RGCs), as well as the retinal nerve fiber layer (RNFL), are severely damaged in AD patients [13,14,15]. Multiple studies have reported a correlation between progressive retinal thinning in AD patients and the severity of cognitive decline [16,17,18]. Furthermore, transgenic AD mouse models, particularly APP/PS1 mice, exhibit similar retinal pathological features, including Aβ plaques, microglial activation, and neuronal degeneration, confirming their value in investigating visual system alterations in AD [19].

However, the visual pathway comprises not only the retina but also multiple interconnected structures such as the V1 and the LGN, together forming a complex network for visual information processing. Visual abnormalities may result from the combined influence of these structures. At present, little is known about the involvement of the LGN in AD progression; a few studies have found only limited Aβ pathology in the LGN [20], and evidence of LGN degeneration in AD remains inconclusive [21]. Studies using 5xFAD mouse models have also reported that LGN function and structure remain relatively stable [22]. Research on V1 has focused more on functional studies, which suggest that early AD disrupts fine visual processing in V1, leading to visual abnormalities [23]. Aβ plaques induce degeneration in V1, thereby affecting neuronal activity and visual tuning. Studies in AD animal models reveal that before amyloid plaques emerge, visual tuning remains intact, but it progressively deteriorates as amyloid burden increases—for example, neuronal tuning to the direction of visual stimuli gradually declines [23]. Alterations in V1 neuronal activity often precede overt amyloid pathology, and as plaques accumulate, spontaneous neuronal activity changes, followed later by reduced overall neuronal activity. These changes in spontaneous activity are often associated with Aβ plaques [24]. In the visual cortex, hyperactive neurons tend to cluster near amyloid plaques (<60 µm), whereas silent and normal neurons are distributed randomly. In later disease stages, overall neuronal activity in the visual cortex tends to be reduced [25].

Despite these advances, several critical knowledge gaps remain in our understanding of degeneration along the visual pathway in AD. First, most studies primarily focus on retinal changes, with a lack of systematic investigations into pathological progression across the entire visual pathway [19,22]. Second, existing studies often probe only a single time point or a limited set of pathological markers, failing to capture the dynamic characteristics of neurodegeneration across disease stages. Third, the specific cell types underlying regional vulnerability within the visual pathway have not been comprehensively characterized. Parvalbumin-positive (PV^+^) neurons, a core pathological component driving cognitive decline and network dysfunction [26], also constitute the most abundant subtype of GABAergic inhibitory neurons in the primary visual cortex (V1) and play a key role in visual information processing [27]; nevertheless, their vulnerability over the course of AD remains unclear. These limitations impede the development of visual biomarkers for early AD detection.

To this end, this study investigated pathological and structural changes across multiple components of the visual pathway in APP/PS1 mice at different stages of degeneration. We determined distinct stages of progression through behavioral assessments of memory function. Using immunohistochemistry, we examined the V1, LGN, and retina, at 3, 6, and 9 months of age, corresponding, respectively, to the pre-symptomatic, early symptomatic, and progressive stages of hippocampal pathology. By correlating immunohistochemical analyses of Aβ distribution, microglial activation, and neuronal loss with structural measurements of the visual cortex and retina, we aim to establish the spatiotemporal pattern of degeneration along the visual pathway and its relationship to the progression of cognitive decline.

Ultimately, our objective was to characterize pathological changes at multiple time points in three key structures of the visual pathway (the retina, LGN, and V1) during AD progression in APP/PS1 mice, thereby delineating a region-specific vulnerability pattern. By correlating structural/pathological alterations with behavioral assessments of long-term and working memory, we established the temporal relationship between degeneration of the visual pathway and the progression of cognitive decline.

## 2. Materials and Methods

### 2.1. Mice

The APP/PS1 mouse line was generated by introducing human amyloid precursor protein (APP) and presenilin-1 (PS1) transgenes into the mouse genome using transgenic techniques. It expresses human familial Alzheimer’s disease (FAD) mutant APP (KM670/671NL, Swedish) and PS1(deltaE9). Experimental APP/PS1 mice were obtained by crossing heterozygous transgenic mice with C57BL/6 wild-type breeders, with wild-type (WT) littermates used as the control group. All experimental procedures were conducted in a randomized and double-blinded manner. All experimental procedures employed male mice, which were purchased from Cavens Biogle (Suzhou) Model Animal Research Co., Ltd. (Suzhou, China). The animals were housed in a standard animal facility under a 12 h light/dark cycle and provided food and water ad libitum. The animals are raised in groups to meet their social needs. At the same time, they are kept in a cage of 2 to 4 animals each to prevent overcrowding which could lead to fights among the animals. Nesting materials such as sterilized paper cotton, gnawing blocks, and plastic shelter houses are provided in the cages to promote the expression of their natural behaviors. Data were collected at 3-month intervals (i.e., at 3–9 months) from APP/PS1 mice and their wild-type littermates. All animal experiments were conducted in accordance with the approval of the Animal Care and Use Committee of the University of Electronic Science and Technology of China. All behavioral tests were conducted at the same time of day to maintain consistency and minimize potential influences of the animals’ circadian rhythms (9:00 A.M.–12:00 P.M.). The order of mouse testing was randomized across experimental groups to prevent sequential bias. Furthermore, throughout the processes of behavioral and histological staining assessments as well as data analysis, the experimenters were blinded to the group identities of the animals (e.g., genotype or months).

### 2.2. Behavioral Assessments

#### 2.2.1. Novel Object Recognition

**Figure 1:**
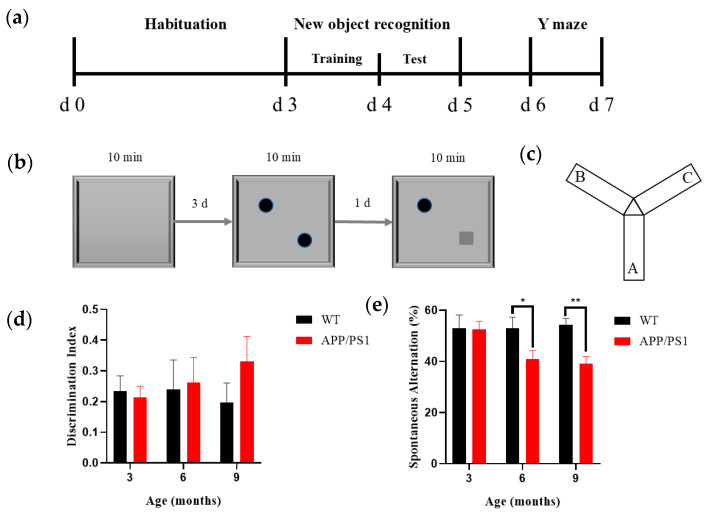
Cognitive function assessment of 3-, 6-, and 9-month-old WT and APP/PS1 mice (*n* = 8/group) (**a**) Schematic diagram of the experimental procedure for behavioral testing. (**b**) Diagram of New object recognition (NOR) testing. (**c**) Diagram of Y maze testing. (**d**) Discrimination index of NOR testing in 3-, 6-, and 9-month-old WT mice (black) and the same-month-old APP/PS1 mice (red). No significant change in the discrimination index was observed in either group of mice (*n* = 8 per group, per time-point). (**e**) Spontaneous alternation rate of Y maze testing in 3-, 6-, and 9-month-old WT mice (black) and the same-month-old APP/PS1 mice (red). *n* = 8 per group, per time-point. * *p* < 0.05, ** *p* < 0.01.

Mice underwent NOR behavioral assessments at 3, 6, and 9 months of age. The NOR apparatus comprised a testing box, objects, and a behavioral recording/analysis system; the box was an open-topped rectangular arena (40 cm × 40 cm × 40 cm; L × W × H) made of odorless white medical-grade acrylic. The objects were an odorless, smooth orange-red cylinder (diameter 3 cm, height 3 cm) and a green cube (edge length 3 cm), affixed along the diagonal of the arena so that they could not be moved by the mice or climbed upon (Figure 1b). Recording and analysis were performed using the SMART v3.0 small animal behavioral system (Panlab, Barcelona, Spain); the apparatus was placed in a quiet room with constant illumination (100 lux), and the NOR test consisted of three phases: habituation, training, and testing. During the habituation phase (3 days), each mouse was placed individually into the empty box to explore freely for 10 min. Twenty-four hours after the habituation phase, the training session was conducted, with two identical objects placed evenly along the diagonal of the box; each mouse was placed individually into the box to explore freely for 10 min. 24 h after the training phase, the test session was conducted, in which one object was replaced with a novel object differing in shape and color but matched in size; each mouse was placed individually into the box to explore freely for 10 min while behavior was recorded by the analysis system. Between experimental phases and between the tasks of any two mice, the objects and box were wiped with 75% ethanol to minimize olfactory interference with free exploration behavior. The experimental design workflow for the novel object recognition test is shown in Figure 1b. Results were presented using the discrimination index (DI), a commonly used metric for distinguishing between familiar and novel objects (1) [28]. The formula for its calculation is as follows:

(1) $$DI=\frac{{Time}_{novel\;location}-{Time}_{familiar\;location}}{{Time}_{novel\;location}+{Time}_{familiar\;location}}$$

#### 2.2.2. Y Maze

Following completion of the NOR test, mice underwent the Y-maze test (Figure 1c). The Y-maze apparatus consisted of a Y-maze chamber and a behavioral recording/analysis system, and the maze itself comprised three identical arms constructed from odorless, medical-grade white acrylic. The maze had three arms separated by 120°; each arm measured 30 cm × 8 cm × 15 cm (L × W × H), and distinct geometric shapes were affixed inside each arm as visual cues. Recording and analysis were performed using the SMART small animal behavioral system (v3.0, Panlab, Spain); the Y-maze was placed in a quiet room with constant illumination (100 lux), mice were introduced at the end of the same designated arm, and the sequence of arm entries over a 5 min period was recorded; after each trial, odors were eliminated with ethanol and the next trial commenced after the scent had dissipated. Results were expressed as the spontaneous alternation percentage (SAP) to assess spatial working memory in rodents. An alternation was defined as successive entries into three different arms on overlapping triplet sets of choices (e.g., 1-2-3 or 1-3-2). The maximum possible alternations corresponded to the total number of arm entries minus two (2). The calculation formula is as follows:

(2) $$SAP\left(\%\right)=\frac{Number\;of\;actualal\;ternations}{Number\;of\;possible\;alternations}\times 100\%$$

### 2.3. Immunohistochemical Staining

Mice from different groups at different (3, 6, and 9) months of age were anesthetized by intraperitoneal injection of an overdose of sodium pentobarbital; after achieving deep anesthesia, they were euthanized by transcardial perfusion and tissues were collected. Following perfusion, the mouse brain and ipsilateral eyeball (right side) were rapidly removed. The dissected brain tissue was immediately placed in neutral buffered formalin (PH0996, Phygene, Fuzhou, China) for overnight fixation; at the same time the following day, the tissue was removed from PFA, rinsed 3 times in phosphate-buffered saline (PBS), and dehydrated in ethanol, and then the brain and eyeball were infiltrated with paraffin, embedded, and labeled. Mouse brains were sectioned symmetrically in the coronal plane and eyeballs were sectioned symmetrically along the optic nerve (Microtome, HM340E, Thermo Fisher Scientific, Waltham, MA, USA), and paraffin sections were cut at a thickness of 4 μm. Brain section sampling: Brains were paraffin-embedded and coronally sectioned at 4 μm. Every 5th section (20 μm) was analyzed, starting from the first section meeting the anatomical boundaries of V1, dorsal LGN, and CA1 as defined by a standard brain atlas (e.g., Paxinos & Franklin). For each stain, three sections per region per animal were quantified. Analyses were blinded. Retina sampling: Eyes were paraffin-embedded and sagittally sectioned at 4 μm. Only sections that contained the optic nerve head (ONH) were included. We applied systematic sampling within the eligible sequence and analyzed every other section (8 μm), starting from the first eligible section; for each stain, three ONH-containing sections per eye were quantified. Analyses were blinded. After deparaffinization and rehydration, brain and retinal sections were washed 3 times with PBS, blocked for 2 h in PBS containing 10% goat serum and 0.3% Triton X-100, and then incubated overnight with primary antibodies diluted in blocking buffer (PBS supplemented with 5% normal goat serum and 0.3% Triton X-100). Sections incubated overnight were washed three times with PBS and then incubated with secondary antibodies diluted in blocking solution (PBS containing 5% normal goat serum and 0.3% Triton X-100) for 2 h at room temperature in the dark (in a light-proof box). Subsequently, sections were washed three times with PBS to remove residual secondary antibodies, mounted with a medium containing DAPI, and rinsed with double-distilled water. Immunofluorescence staining was used to examine Aβ and microglia in the visual pathway and hippocampal regions, to label neurons in V1 and the LGN, and to identify PV neurons in V1. Fluorescence images were obtained using a slide scanner (3D-Histech, Pannoramic MIDI II, Budapest, Hungary). The primary antibodies used were as follows: rabbit anti-Aβ (CST, #8243,1:500, Danvers, MA, USA), rabbit anti-Iba-1 (Abcam, ab178847,1:200, Cambridge, UK), rabbit anti-NeuN (Abcam, ab177487, 1:500, Cambridge, UK), and mouse anti-PV (Synaptic Systems, 195011, 1:500, Göttingen, Germany). The secondary antibodies included Alexa Fluor 594 goat anti-rabbit (Invitrogen, A-11012, 1:500, Waltham, MA, USA) and Alexa Fluor 488 goat anti-mouse (Invitrogen, A-11001, 1:500, Waltham, MA, USA).

### 2.4. Hematoxylin–Eosin Staining

Brain and retinal sections were washed 3 times in PBS, then stained with hematoxylin for 3 min; sections were rinsed under running double-distilled water to remove excess stain, followed by differentiation in a differentiation solution for 5 s to control staining depth. Subsequently, sections were blued with a bluing reagent for 1 min to enhance staining contrast. The sections were then rinsed. The processed retinal sections were stained with eosin for 20 s. Subsequently, graded dehydration was performed (80% ethanol for 2 s, followed by 95% ethanol for 1 min), after which sections were placed in absolute ethanol I and absolute ethanol II for 60 s each. Finally, sections were cleared in xylene I and xylene II for 2 min each. After air-drying, sections were coverslipped using a neutral balsam mounting medium. Bright-field images were captured using the same slide scanner as for fluorescence imaging.

### 2.5. Data Analysis

Quantitative analysis of immunohistochemistry images was performed using ImageJ (1.54g, NIH, Bethesda, MD, USA). For Aβ quantification, images were captured using identical microscope settings and analyzed using ImageJ software (NIH). All images were converted to 8-bit grayscale. The threshold for Aβ-positive staining was determined from wild-type control sections across all age groups (mean background intensity + 2 standard deviations) and was held constant across all images. The Aβ-positive area was calculated as the percentage of pixels above the threshold. The sections per region per animal were analyzed for cell counting, with three random non-overlapping fields selected from standardized anatomical sites. Cells were quantified using ImageJ with uniform thresholds. Double-blind counting by two independent investigators yielded averaged results expressed as cells/mm^2^. Behavioral data from the novel object recognition and Y-maze tests were analyzed using SMART v3.0. All quantitative data are presented as mean ± standard error of the mean (Mean ± SEM). All statistical analyses and graphing were performed using GraphPad Prism 8.0 (GraphPad Software, La Jolla, CA, USA). Prior to parametric testing, data normality was assessed with the Shapiro–Wilk test and homogeneity of variance with Levene’s test. Two-way ANOVA was employed to analyze differences in group means when both normality and homoscedasticity assumptions were satisfied (group ×months). If significant effects were identified, post hoc pairwise comparisons were conducted using the Tukey test. For data not meeting these assumptions, the Kruskal–Wallis nonparametric test followed by Dunn’s multiple comparisons was applied. Statistical significance was considered at *p* < 0.05.

## 3. Results

### 3.1. Early Impairment of Spatial Working Memory and Relative Preservation of Recognition Memory in APP/PS1 Mice

To delineate distinct stages of AD pathology and provide a functional basis for subsequent analyses of pathological and neurostructural features, we employed two classical behavioral paradigms—the NOR test and the Y-maze spontaneous alternation test—to assess long-term recognition memory and spatial working memory, respectively, in experimental animals. By tracking the extent and temporal dynamics of impairments in these two memory systems, we established the correspondence between cognitive decline and disease progression, thereby enabling staging of the AD course.

NOR results showed that wild-type (WT) mice maintained a stable discrimination index (DI) between 3 and 9 months of age, with no age-related decline (Figure 1d, black bars). In APP/PS1 transgenic mice, the DI at 9 months did not differ significantly from age-matched WT controls (*p* > 0.05), nor from their own 3-month baseline (Figure 1d, red bars), indicating that long-term recognition memory was relatively preserved over the observation period.

Y-maze results indicated that the spontaneous alternation percentage (SAP) in WT mice remained constant throughout the observation period (Figure 1e). By contrast, a significant reduction in SAP was observed in APP/PS1 mice starting at 6 months of age (*p* < 0.05 vs. age-matched WT), which further worsened by 9 months, suggesting a progressive trajectory of spatial working memory deficits.

Collectively, these findings indicate domain-specific and temporal characteristics of cognitive impairment in APP/PS1 mice, with spatial working memory deficits emerging earlier than recognition memory deficits, thereby providing behavioral evidence for staging the AD course.

### 3.2. Comparative Analysis of Aβ Plaque Distribution Along the Visual Pathway at Different Ages in APP/PS1 Mice

Aβ occupies a central position in the pathogenesis of AD. Extensive evidence has established that pathological aggregation of Aβ initiates the AD cascade, triggering multiple neurotoxic events—including oxidative stress, neuroinflammation, and synaptic dysfunction—that ultimately lead to progressive neurodegeneration. As the disease advances, affected brain regions exhibit pronounced neuronal loss, clinically manifesting as progressive declines in cognitive and perceptual functions. Building on this pathological basis, cohorts at 3, 6, and 9 months of age (*n* = 6–7 per group) were included, and frozen sections of brain and eyeball were prepared. Using Aβ immunofluorescence, we systematically evaluated Aβ plaque distribution in the V1, LGN, retina, and hippocampus to delineate the spatiotemporal evolution of Aβ pathology across the visual pathway during AD progression.

**Figure 2:**
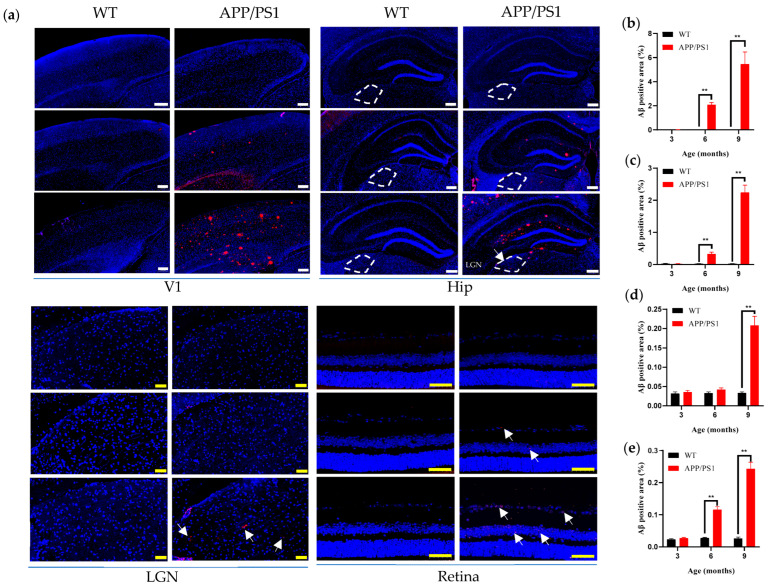
Aβ distribution in visual pathway and hippocampus of 3-, 6- and 9-month-old WT and APP/PS1 mice. (**a**) Immunofluorescent staining for Aβ (red) in the Visual pathway and Hippocampus of mice (WT group, APP/PS1 group) at 3, 6, and 9 months of age. Scale bar, 200 μm (white), 50 μm (yellow). (**b**) Percentage of positive area of Aβ plaque per mm^2^ in V1 for WT group (black), APP/PS1 group (red). (**c**) Aβ plaque area per mm^2^ in the Hippocampus for WT group (black), APP/PS1 group (red). (**d**) Aβ plaque area per mm^2^ in the LGN for WT group (black), APP/PS1 group (red). (**e**) Aβ plaque area per mm^2^ in the Retina for WT group (black), APP/PS1 group (red). The LGN region is outlined by a white dotted line. Red indicates Aβ plaques, and arrows point to representative plaques. *n* = 6–7 per group. ** *p* < 0.01.

The results (Table 1) showed that no amyloid plaques were detected in any examined region of WT mice aged 3–9 months (Figure 2a; Figure 2b–d, black bars). Compared with WT controls, amyloid plaque deposition was detectable in V1, the retina, and the hippocampus of AD model mice by 6 months of age (Figure 2a), with plaque burden increasing in an age-dependent manner (Figure 2a–c) and peaking at 9 months. Notably, the onset of Aβ plaques in V1 and the retina was synchronous with that in the hippocampus, and all three regions exhibited time-dependent accumulation of amyloid deposition. By contrast, pathological changes in the LGN appeared relatively delayed: no amyloid plaques were detected at 6 months, whereas a clear plaque distribution became apparent at 9 months (Figure 2d). Using 6 months as the reference time point, regional plaque burden followed the pattern V1 (2.09 ± 0.17, ~2%) > hippocampus (0.33 ± 0.05, ~0.33%) > retina (0.12 ± 0.01, ~0.12%) (Figure 2a–c,e). Moreover, the results showed that the amyloid burden in the V1 region was significantly greater than in the hippocampus and retina at both 6 and 9 months of age (Figure 4a).

### 3.3. Comparative Analysis of Microglial Proliferation Along the Visual Pathway at Different Ages in APP/PS1 Mice

Pathological accumulation of β-amyloid (Aβ) can elicit multiple neurotoxic responses, among which microglial activation is a key pathological feature. Previous studies have shown that activated microglia mediate synaptic loss via complement-dependent synaptic pruning [29,30,31], thereby leading to neuronal dysfunction. Given the heterogeneity in the spatiotemporal distribution of amyloid plaques across the visual pathway, we hypothesized that microglial activation patterns would exhibit corresponding region-specific and temporal differences. To test this, we systematically assessed microglial activation across brain regions using immunostaining for the microglial marker Iba-1.

**Figure 3:**
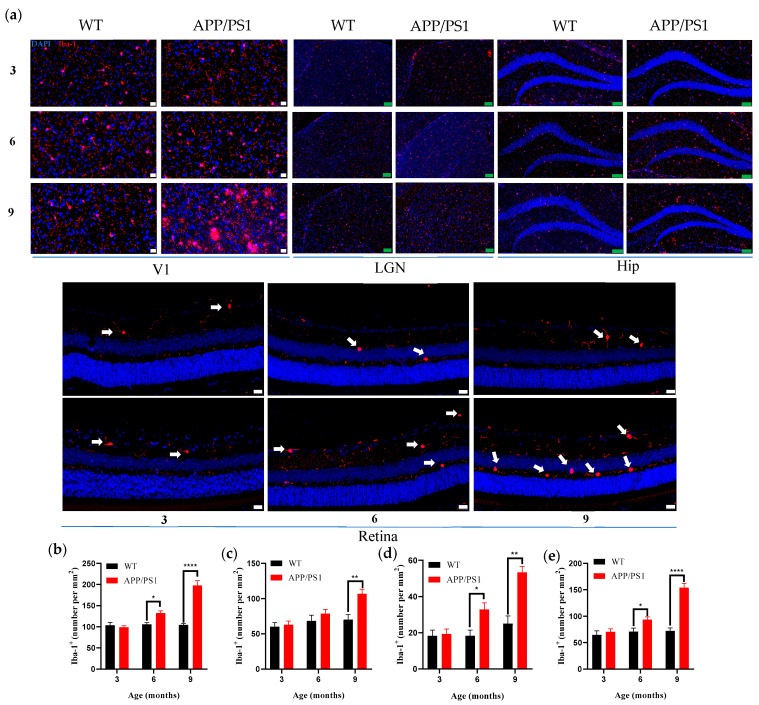
Microgliosis in Visual pathway and Hippocampus from 3-, 6- and 9-month-old WT and APP/PS1 mice. (**a**) Immunofluorescent staining for Iba-1 (red) in the Visual pathway and Hippocampus of mice (WT group, APP/PS1 group) at 3, 6, and 9 months old. Scale bar, 20 μm (white), 100 μm (green). (**b**) Iba-1^+^ positive cell density (cell per mm^2^) in V1 for WT group (black), APP/PS1 group (red). (**c**) Iba-1^+^ positive cell density (cell per mm^2^) in LGN for WT group (black), APP/PS1 group (red,). (**d**) Iba-1^+^ positive cell density (cell per mm^2^) Retina for WT group (black), APP/PS1 group (red). (**e**) Iba-1^+^ positive cell density (cell per mm^2^) in Hippocampus for WT group (black), APP/PS1 group (red). Iba-1 immunoreactivity (red) labels microglia, and DAPI (blue) stains nuclei. Arrows point to microglial cells in the retina. *n* = 6–7 per group. * *p* < 0.05, ** *p* < 0.01, **** *p* < 0.0001.

**Figure 4:**
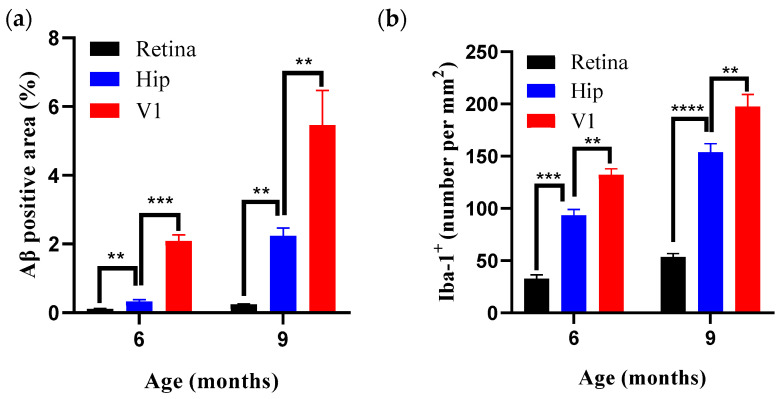
Region- and age-dependent differences in Aβ plaque burden and microgliosis in APP/PS1 mice. (**a**) Aβ plaque burden in APP/PS1 mice across different brain regions (V1, Hippocampus, Retina) and ages (6- and 9-month). (**b**) microgliosis in APP/PS1 mice across different brain regions (V1, Hippocampus, Retina) and ages (6- and 9-month). *n* = 7 per group. ** *p* < 0.01, *** *p* < 0.001, **** *p* < 0.0001.

Results (see Table 1) showed that in wild-type mice, microglial density in nuclei along the primary visual pathway and in the hippocampus remained stable from 3 to 9 months, with no significant age-related changes (Figure 3a; Figure 3b–e, black bars). Consistent with the pattern of Aβ pathology, APP/PS1 mice showed a significant increase in the density of Iba-1–positive cells in V1 and the hippocampus beginning at 6 months (* *p* < 0.05, vs. age-matched WT), with a clear age-dependent upward trajectory (Figure 3a; Figure 3b,e, red bars). Notably, the microglial response in the LGN was relatively delayed, with a significant increase in Iba-1–positive cells observed only at 9 months (Figure 3a,c). The retina likewise exhibited increased microglial density at 6 months, which progressed further with disease advancement (Figure 3a,d). Furthermore, the results showed that the proliferation of microglia in the V1 region was significantly higher than in the hippocampus and retina at both 6 and 9 months of age (Figure 4b).

### 3.4. No Changes in Retinal Thickness and V1 Cortical Thickness in APP/PS1 Mice

**Figure 5:**
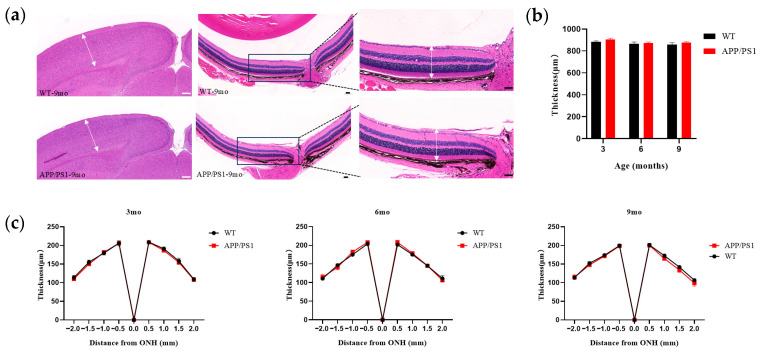
Thickness of V1 and Retina from 3-, 6- and 9-month-old APP/PS1 and WT mice. (**a**) HE staining in V1 and Retina of mice (WT group, APP/PS1 group) at 9 months of age. Scale bar, 200 μm (white), 50 μm (black). (**b**) Thickness of V1 for WT group (black), APP/PS1 group (red). (**c**) Retinal thickness for WT group (black), APP/PS1 group (red). *n* = 6–7 per group. The white arrow indicates the thickness (V1, Retina), and the box represents the magnified view of this position (Retina).

The above results indicate that pathological alterations emerge relatively early in V1 and the retina within the primary visual pathway during the AD course. To determine whether these pathological changes are accompanied by structural alterations, we employed HE staining to perform morphological assessments of the V1 cortex and retina in APP/PS1 transgenic mice and wild-type controls aged 3–9 months, focusing on thickness changes. The results showed no evident atrophic changes in the V1 cortex of APP/PS1 mice; cortical thickness did not differ significantly from age-matched WT controls (Figure 5b). For retinal thickness evaluation, we measured total retinal thickness. Given physiological regional differences, to ensure accuracy and reproducibility we used the optic disc as a reference point and measured full-thickness retina at 0.5, 1.0, 1.5, and 2.0 mm on either side of the disc. Morphometric analysis indicated that retinal thickness in APP/PS1 mice showed no significant disease-related changes at any measured site and did not differ statistically from age-matched controls (Figure 5c).

### 3.5. Differential Vulnerability Patterns of Neurons in V1 and LGN in APP/PS1 Mice

**Figure 6:**
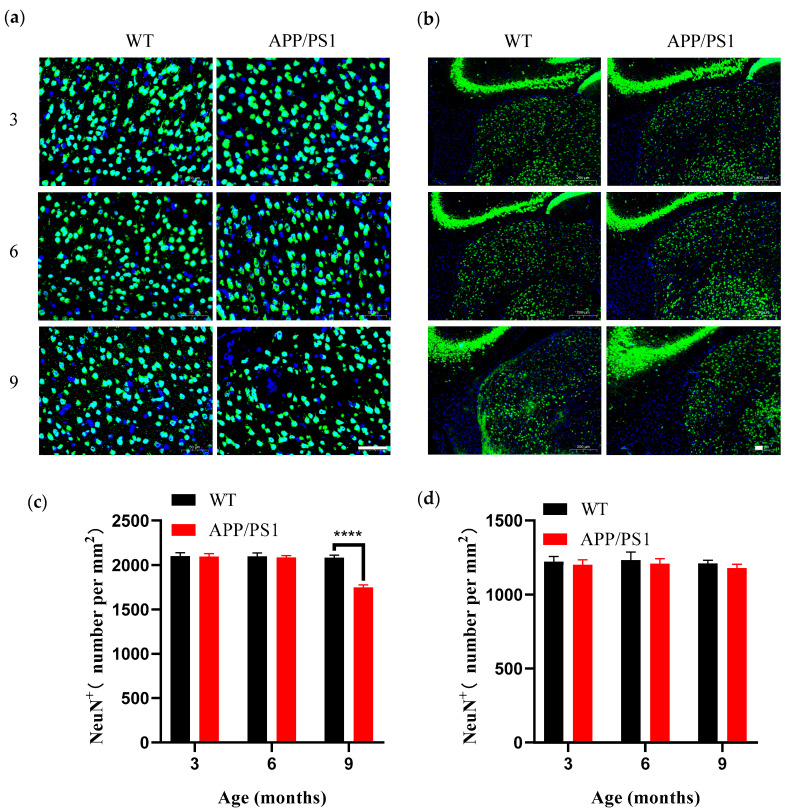
Number of neurons in V1 and LGN from 3-, 6- and 9-month-old WT and APP/PS1 mice (**a**,**b**) Immunofluorescent staining for NeuN (green) and DAPI (blue) in the V1 and LGN of mice (WT group, APP/PS1 group) at 3, 6, and 9 months of age. Scale bar, 50 μm. (**c**) Number of NeuN^+^ per mm^2^ in the V1 for WT group (black), APP/PS1 group (red). (**d**) Number of NeuN^+^ per mm^2^ in the LGN for WT group (black), APP/PS1 group (red). *n* = 6–7 per group. **** *p* < 0.0001.

Building on the above finding that V1 exhibits selective vulnerability within the visual pathway, we further investigated differential pathological vulnerability among neuronal subpopulations in V1. We used the neuronal nuclear marker NeuN (neuronal nuclei) to assess overall neuronal density, which primarily reflects changes in excitatory pyramidal neurons that constitute about 80% of cortical neurons. In parallel, given that parvalbumin-positive (PV^+^) interneurons are the most abundant subtype of GABAergic interneurons (~40–50%) and play a critical role in maintaining cortical network function, we chose PV as a representative interneuron marker to evaluate how inhibitory neurons respond to AD pathology. Immunohistochemical analyses showed no age-related neuronal loss in WT mice between 3 and 9 months of age. However, in 9-month-old APP/PS1 mice, A significant reduction in NeuN^+^ neuronal density (cells/mm^2^) was observed in the V1 of APP/PS1 mice compared with WT controls (Figure 6a,b), indicating that when amyloid plaque burden reaches a high level (>5% of cortical area), V1 undergoes marked neuronal loss, a change that largely reflects damage to the predominant pyramidal neuron population. By contrast, no corresponding decrease in neuronal density was observed in the LGN of APP/PS1 mice (Figure 6c), corroborating the region-specific nature of neurodegeneration.

### 3.6. Differential Vulnerability Patterns Among Major Interneuron Subpopulations in V1 of APP/PS1 Mice

**Figure 7:**
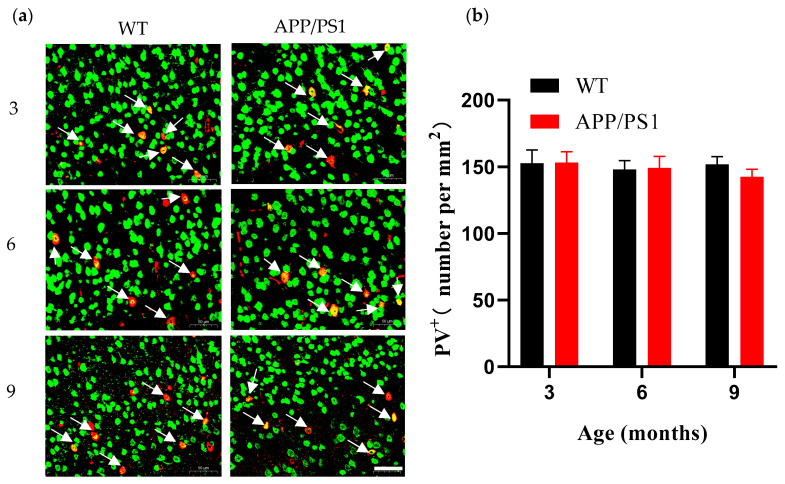
Number of PV^+^ neurons in V1 from 3-, 6- and 9-month-old WT and APP/PS1 mice (**a**) Immunofluorescent staining for PV^+^ neuron (red) and NeuN^+^ (green) in the V1 of mice (WT group, APP/PS1 group) at 3, 6, and 9 months of age. The white arrow points to PV^+^ neurons identified by NeuN^+^ colocalization, Scale bar, 50 μm. (**b**) Number of PV^+^ neurons per mm^2^ in V1 of WT mice (black, *n* = 6) and APP/PS1 mice (red, *n* = 7).

Analysis of PV^+^ interneuron subpopulations revealed that PV^+^ neuron density remained stable in WT mice throughout the observation period. Importantly, even in 9-month-old APP/PS1 mice exhibiting a high amyloid plaque burden (>5%), the number of PV^+^ interneurons in V1 remained relatively stable (Figure 7a,b). These findings suggest that the overall decline in neuronal density observed in V1 primarily reflects loss of excitatory pyramidal neurons, whereas PV^+^ interneurons—the predominant inhibitory neuronal subtype—exhibit relative resistance to AD-related pathology; such differential vulnerability between excitatory and inhibitory neurons may lead to cortical network imbalance.

## 4. Discussion

Alterations in the visual system and retinal pathology are emerging biomarkers for predicting the early course of Alzheimer’s disease (AD). However, some studies suggest that early visual deficits in AD may stem from cortical impairments in visual information processing [23]. At present, conclusions about the origin of visual abnormalities are contradictory, and the temporal sequence of pathological events along the visual pathway remains unclear. Accordingly, clarifying stage-specific changes in the primary visual pathway—particularly those surrounding the emergence of amyloid plaques—will be crucial for understanding how pathology develops and evolves.

Using behavioral assessment methods, we staged disease progression in APP/PS1 mice, thereby providing a clear temporal framework for pathological analyses. Using immunohistochemical techniques, we systematically characterized features of pathological progression at different disease stages. The study focused on pathological and structural alterations of the primary visual pathway in 3–9-month-old APP/PS1 mice and their WT controls across three key stages—prior to amyloid plaque formation, at plaque onset, and during plaque enrichment.

We found no significant decline in the recognition index of long-term memory in APP/PS1 mice from 3 to 9 months, nor any significant increase in WT mice at the same time points; notably, a meta-analysis [32] reported no significant association between experimentally assessed cognitive deficits and quantified brain Aβ levels, which our findings corroborate. Our results further indicate that spatial memory behavior did not decline even by the stage of abundant hippocampal plaques, consistent with prior reports [19]. By contrast, spatial working memory in APP/PS1 mice showed a reduction in spontaneous alternation beginning at 6 months and worsening by 9 months, suggesting that 6 months marks a critical time point at which the encoding and short-term storage of spatial information become impaired.

We observed that pathology within the primary visual system (V1–LGN–retina) precedes hippocampus-mediated cognitive-behavioral changes, with V1 exhibiting a pattern similar to the retina. Within the primary visual system, V1 and the retina exhibited earlier pathological changes—specifically, earlier amyloid pathology, both detectable at 6 months—suggesting a potential synchrony between the emergence of retinal Aβ plaques and brain plaques during disease progression; notably, one study reported retinal Aβ plaques at 2.5 months in APP/PS1 mice [12], 2–3 months earlier than in the brain, a discrepancy that may reflect methodological differences in Aβ labeling (curcumin-based probes visualizing Aβ and its isomers in that study versus conventional Aβ antibodies in ours). V1 exhibited greater pathological involvement (by concurrent plaque burden) than the hippocampus, yet its structure—cortical thickness of V1—remained unchanged even at high plaque burden. There remains some controversy regarding changes in retinal thickness. One report in 5 × FAD mice found no change in total retinal thickness up to 12 months, whereas another reported significant differences in inner retinal layer thickness between APP/PS1 and WT mice at 9 months [19]; in our study, total retinal thickness did not change up to 9 months, and the discrepancy may arise because we measured overall retinal thickness, with outer layers potentially offsetting inner-layer differences, suggesting that 9 months may be a key time point for structural alterations in the retina. Concurrently, both V1 and the retina displayed microglial proliferation coincident with plaque emergence at 6 months; such proliferation indicates a persistent inflammatory response in the brain and suggests a relatively early and possibly synchronous degeneration of the retina and brain. In the course of AD, LGN pathology emerges relatively late; the LGN, a key dorsal thalamic structure, primarily relays visual information by transmitting signals from the retina to V1, thereby enabling efficient flow and processing of visual input. We found that the LGN was relatively stable across the primary visual pathway: even when V1 had abundant plaques (9 months), the LGN showed no neuronal loss and only a few small amyloid plaques. This is consistent with observations from postmortem studies.

V1 contains a large diversity of neuronal types that encode and decode visual information and serves as a central hub for visual processing. Loss of neurons is another prominent feature of AD pathology, contributing to cognitive impairment and abnormalities in the visual cortex. As the processing center for visual information, V1 relies on interactions among distinct neuronal types to accomplish visual decoding. Loss of PV^+^ neurons can disrupt visual coding; PV^+^ neurons are considered critical for encoding visual perception in V1, and studies show that enhancing PV^+^ neuron activation improves V1 coding and perception [27]; PV^+^ neurons also play a major role in generating gamma-band oscillations, a characteristic frequency band of normal neuronal activity. However, the frequency of gamma oscillations is reduced in the brains of patients with neurodegenerative diseases [33], a phenomenon likewise observed in AD transgenic mice [26,33]. We observed neuronal loss at the stage of abundant plaques (9 months), yet the PV^+^ neurons did not decline in V1 despite the high plaque burden, in contrast to the loss observed in the overall neuronal population. Reports on PV^+^ neurons are mixed, ranging from substantial loss to no change in cell number [34,35,36,37,38]; for example, immunofluorescence in 9–11-week-old APP/PS1 mice revealed a significant reduction of PV^+^ interneurons in the PFC [39], suggesting regional specificity in PV^+^ neuron alterations: PV^+^ neurons in V1 appear resistant, showing no loss even at high plaque burden, and damage to PV^+^ neurons in the primary visual cortex may occur relatively late. Functional changes in V1 neurons are thought to arise from differing susceptibilities of neuronal types as plaque burden increases, reflecting the combined effects across cell classes. Damage to inhibitory interneurons can produce connectivity defects, leading to aberrant network activity and cognitive impairment [26]. A study in the hippocampus of AD mice found differential sensitivity of neuronal types (pyramidal vs. interneurons) to amyloid: PV neurons were hyperactive before plaque emergence, thereby suppressing pyramidal neuron activity, whereas after amyloid deposition PV hyperactivity was dampened, leading to pyramidal neuron hyperactivity [40,41]. In APP/PS1 mice, hippocampal PV^+^ neurons dysfunction occurs at early AD stages [41]; does this amyloid sensitivity of PV neurons generalize across regions? PV^+^ neurons express specific ion channel subunits, such as Nav1.1 and Kv3, which underlie their fast-spiking properties; thus, their apparent resistance may reflect direct Aβ effects on PV neuronal channels or synapses rather than frank loss of PV neurons. How PV neurons in V1 change in function and structure in response to amyloid during AD progression will be a key focus of our subsequent investigations. A noteworthy finding in the present study is the significant reduction in V1 neuronal density at 9 months without a corresponding decrease in total V1 cortical thickness. This dissociation between reduced neuronal density and maintained cortical thickness suggests several potential compensatory mechanisms. First, the remaining neurons may undergo compensatory somatic enlargement. Recent studies have demonstrated that microglia in the peripheral nervous system can directly regulate neuronal soma size. Under pathological conditions in the central nervous system, activated microglia surrounding surviving neurons may promote compensatory enlargement of remaining neurons through similar mechanisms [42]. Second, microglial proliferation may be a critical factor in maintaining cortical thickness. Reactive glial proliferation represents a typical response following neuronal injury, with microglial proliferation and aggregation potentially filling the space left by neuronal loss [43]. Third, neural network remodeling may also contribute to cortical structural maintenance. Despite reduced neuronal numbers, surviving neurons may undergo compensatory dendritic branching and axonal growth, forming more complex neural networks to preserve function [44]. While cortical thickness is maintained, the reduction in neuronal density may still result in impaired visual information processing capacity.

This study reveals the central role of V1 in visual pathway damage. As a critical hub for visual information processing, progressive neuronal loss in V1 accompanied by microglial proliferation may constitute the important pathological basis for functional deficits. To gain deeper insights into the functional significance of these structural changes, future research should integrate electrophysiological and behavioral assessment approaches.

Visual evoked potentials (VEPs), as a non-invasive technique that records brain responses to visual stimuli through electroencephalography, provide an ideal avenue for functional assessment. The reduction in V1 neuronal density and consequent decline in signal processing efficiency should theoretically manifest as abnormal VEP parameters, such as prolonged latency or reduced amplitude of the P100 component. Given the cost-effectiveness, operational simplicity, and non-invasive safety of VEP examination, it holds excellent clinical application prospects for development as an early functional abnormality screening tool.

The Y-maze spontaneous alternation task employed in this study primarily assesses hippocampus-related spatial working memory function. Although animal experimental paradigms cannot be directly transplanted to clinical settings, their core cognitive domain—the capacity for short-term retention and manipulation of spatial information—can be effectively translated into human neuropsychological assessment methods. The Corsi block test, as a classic non-verbal spatial working memory assessment tool, requires subjects to reproduce specific spatial sequences and can quantitatively evaluate visuospatial short-term memory capacity. Virtual reality-based navigation tasks can construct immersive virtual environments requiring subjects to learn and memorize specific pathways, offering good ecological validity and sensitive detection of spatial orientation deficits in early Alzheimer’s disease patients.

A more prospective strategy involves synchronous recording of V1 neural electrophysiological signals with behavioral testing. In animal models, real-time monitoring of V1 single-cell spiking activity and local field potentials during spatial memory task execution can analyze task-related neural network synchrony changes. In clinical translation, simultaneous recording of occipital neural oscillations through high-density electroencephalography, combined with eye-tracking or functional near-infrared spectroscopy techniques, can capture neural electrophysiological changes that precede behavioral abnormalities. This multimodal integration approach, through establishing correlational models between neural electrophysiological markers and cognitive performance, holds promise for discovering more sensitive and mechanistically specific early diagnostic biomarkers, providing crucial technical support for precision diagnosis of Alzheimer’s disease.

Several limitations of this study should be acknowledged. First, we exclusively used male APP/PS1 mice, which may limit the generalizability of our findings. Given the exploratory nature of this study, we selected male mice to minimize potential confounding factors, particularly to avoid the effects of estrous cycle fluctuations on neuroinflammation and cognitive function in female mice. However, considering that Alzheimer’s disease affects both sexes and accumulating evidence indicates sex differences in disease progression, future studies should include female mice to determine whether the spatiotemporal alterations in visual pathways we observed are sex-dependent and to assess their generalizability across sexes. Second, while the APP/PS1 transgenic model successfully recapitulates key features of amyloid pathology, including plaque formation and associated neuroinflammation, it lacks other critical characteristics of human AD, most notably tau pathology and neurofibrillary tangles. This limitation affects the interpretation of the observed neurodegenerative patterns, as tau pathology is more closely correlated with neuronal loss and clinical dementia severity in human AD. Therefore, validation of our findings in tau-expressing models (such as the 3xTg-AD model) or in human postmortem brain tissue samples would enhance the translational value and clinical relevance of this study.

## 5. Conclusions

In APP/PS1 mice, early pathology along the visual pathway is regionally heterogeneous and extends beyond the retina. At 6 months, the V1 and hippocampus exhibit concurrent Aβ deposition and microglial activation, accompanied by selective spatial working memory deficits; analogous changes in the LGN emerge only by 9 months. By 9 months, V1 shows selective neuronal loss with relative preservation of PV-positive neurons, while no neuronal loss is detected in the LGN, and the gross thickness of the retina and V1 remains stable across all time points. These findings suggest that V1 may be a preferentially affected node along the visual pathway in Alzheimer’s disease and could serve as a potential target for elucidating early mechanisms and for developing visual system-related early diagnostic biomarkers and intervention strategies.

## Figures and Tables

**Table 1:** Temporal sequence of Aβ plaque distribution and microglial proliferation.

Region	Aβ Plaque Onset (Months)	Microglial Proliferation (Months)
Retina	6	6
LGN	9	9
V1	6	6
Hip	6	6

## Data Availability

The original contributions presented in this study are included in the article. Further inquiries can be directed to the corresponding authors.

## References

[1] Alzheimer’s Disease International World Alzheimer Report 2024. Global Changes in Attitudes to Dementia.. https://www.alzint.org/resource/world-alzheimer-report-2024/.

[2] Salobrar-García E., de Hoz R., Ramírez A.I., López-Cuenca I., Rojas P., Vazirani R., Amarante C., Yubero R., Gil P., Pinazo-Durán M.D. (2019). Changes in visual function and retinal structure in the progression of Alzheimer’s disease. PLoS ONE.

[3] Polo V., Rodrigo M.J., Garcia-Martin E., Otin S., Larrosa J.M., Fuertes M.I., Bambo M.P., Pablo L.E., Satue M. (2017). Visual dysfunction and its correlation with retinal changes in patients with Alzheimer’s disease. Eye.

[4] Begde A., Wilcockson T., Brayne C., Hogervorst E. (2024). Visual processing speed and its association with future dementia development in a population-based prospective cohort: EPIC-Norfolk. Sci. Rep..

[5] Hong S., Baek S.-H., Lai M.K., Arumugam T.V., Jo D.-G. (2024). Aging-associated sensory decline and Alzheimer’s disease. Mol. Neurodegener..

[6] Alvite-Piñeiro T., López-López M., Regueiro U., Pías-Peleteiro J.M., Sobrino T., Lema I. (2025). Visual Function in Alzheimer’s Disease: Current Understanding and Potential Mechanisms Behind Visual Impairment. J. Clin. Med..

[7] Zhang Y., Wang Y., Shi C., Shen M., Lu F. (2021). Advances in retina imaging as potential biomarkers for early diagnosis of Alzheimer’s disease. Transl. Neurodegener..

[8] Piro-Gambetti B., Krinsky-McHale S., Kovacs C., Handen B., Christian B., Laymon C.M., Minhas D., Luo W., Yoon D.M., Fleming V.L. (2025). Eye tracking as a tool for detecting Alzheimer’s disease in people with Down syndrome. J. Intellect. Disabil. Res..

[9] Mahoney J.R., Ayers E., Verghese J. (2025). Visual-somatosensory integration as a novel behavioral marker of amyloid pathology. Alzheimer’s Dement..

[10] Cheng J., Paracha S.S., Agrawal S., Wu Z., Sung C.-H. (2025). Recent Advances in Visual Dysfunction and Ocular Biomarkers in Neurological Disorders. Eye Brain.

[11] Zhang J., Shi L., Shen Y. (2022). The retina: A window in which to view the pathogenesis of Alzheimer’s disease. Ageing Res. Rev..

[12] Koronyo-Hamaoui M., Koronyo Y., Ljubimov A.V., Miller C.A., Ko M.K., Black K.L., Schwartz M., Farkas D.L. (2011). Identification of amyloid plaques in retinas from Alzheimer’s patients and noninvasive in vivo optical imaging of retinal plaques in a mouse model. Neuroimage.

[13] Jones-Odeh E., Hammond C.J. (2015). How strong is the relationship between glaucoma, the retinal nerve fibre layer, and neurodegenerative diseases such as Alzheimer’s disease and multiple sclerosis?. Eye.

[14] Sivak J.M. (2013). The aging eye: Common degenerative mechanisms between the Alzheimer’s brain and retinal disease. Invest. Ophthalmol. Vis. Sci..

[15] Ko F., Muthy Z.A., Gallacher J., Sudlow C., Rees G., Yang Q., Keane P.A., Petzold A., Khaw P.T., Reisman C. (2018). Association of Retinal Nerve Fiber Layer Thinning with Current and Future Cognitive Decline: A Study Using Optical Coherence Tomography. JAMA Neurol..

[16] Mutlu U., Colijn J.M., Ikram M.A., Bonnemaijer P.W.M., Licher S., Wolters F.J., Tiemeier H., Koudstaal P.J., Klaver C.C.W., Ikram M.K. (2018). Association of Retinal Neurodegeneration on Optical Coherence Tomography With Dementia: A Population-Based Study. JAMA Neurol..

[17] Tao R., Lu Z., Ding D., Fu S., Hong Z., Liang X., Zheng L., Xiao Y., Zhao Q. (2019). Perifovea retinal thickness as an ophthalmic biomarker for mild cognitive impairment and early Alzheimer’s disease. Alzheimers Dement..

[18] Cunha J.P., Proença R., Dias-Santos A., Almeida R., Águas H., Alves M., Papoila A.L., Louro C., Castanheira-Dinis A. (2017). OCT in Alzheimer’s disease: Thinning of the RNFL and superior hemiretina. Graefes Arch. Clin. Exp. Ophthalmol..

[19] Georgevsky D., Retsas S., Raoufi N., Shimoni O., Golzan S.M. (2019). A longitudinal assessment of retinal function and structure in the APP/PS1 transgenic mouse model of Alzheimer’s disease. Transl. Neurodegener..

[20] Leuba G., Saini K. (1995). Pathology of subcortical visual centres in relation to cortical degeneration in Alzheimer’s disease. Neuropathol. Appl. Neurobiol..

[21] Erskine D., Taylor J.P., Firbank M.J., Patterson L., Onofrj M., O’Brien J.T., McKeith I.G., Attems J., Thomas A.J., Morris C.M. (2016). Changes to the lateral geniculate nucleus in Alzheimer’s disease but not dementia with Lewy bodies. Neuropathol. Appl. Neurobiol..

[22] McCool S., Smith J.C., Sladek A., Fan S., Van Hook M.J. (2025). Retinal and thalamic alterations in the 5xFAD mouse model of Alzheimer’s disease. PLoS ONE.

[23] Grienberger C., Rochefort N.L., Adelsberger H., Henning H.A., Hill D.N., Reichwald J., Staufenbiel M., Konnerth A. (2012). Staged decline of neuronal function in vivo in an animal model of Alzheimer’s disease. Nat. Commun..

[24] Targa Dias Anastacio H., Matosin N., Ooi L. (2022). Neuronal hyperexcitability in Alzheimer’s disease: What are the drivers behind this aberrant phenotype?. Transl. Psychiatry.

[25] Busche M.A., Eichhoff G., Adelsberger H., Abramowski D., Wiederhold K.H., Haass C., Staufenbiel M., Konnerth A., Garaschuk O. (2008). Clusters of hyperactive neurons near amyloid plaques in a mouse model of Alzheimer’s disease. Science.

[26] Verret L., Mann E.O., Hang G.B., Barth A.M., Cobos I., Ho K., Devidze N., Masliah E., Kreitzer A.C., Mody I. (2012). Inhibitory interneuron deficit links altered network activity and cognitive dysfunction in Alzheimer model. Cell.

[27] Lee S.H., Kwan A.C., Zhang S., Phoumthipphavong V., Flannery J.G., Masmanidis S.C., Taniguchi H., Huang Z.J., Zhang F., Boyden E.S. (2012). Activation of specific interneurons improves V1 feature selectivity and visual perception. Nature.

[28] Lueptow L.M. (2017). Novel Object Recognition Test for the Investigation of Learning and Memory in Mice. J. Vis. Exp..

[29] Huo K., Wei M., Zhang M., Wang Z., Pan P., Shaligram S.S., Huang J., Prado L.B.D., Wong J., Su H. (2021). Reduction of neuroinflammation alleviated mouse post bone fracture and stroke memory dysfunction. J. Cereb. Blood Flow Metab..

[30] Xu Y., Jiang C., Wu J., Liu P., Deng X., Zhang Y., Peng B., Zhu Y. (2022). Ketogenic diet ameliorates cognitive impairment and neuroinflammation in a mouse model of Alzheimer’s disease. CNS Neurosci. Ther..

[31] Rajendran L., Paolicelli R.C. (2018). Microglia-Mediated Synapse Loss in Alzheimer’s Disease. J. Neurosci..

[32] Zhang R., Xue G., Wang S., Zhang L., Shi C., Xie X. (2012). Novel object recognition as a facile behavior test for evaluating drug effects in AβPP/PS1 Alzheimer’s disease mouse model. J. Alzheimers Dis..

[33] Iaccarino H.F., Singer A.C., Martorell A.J., Rudenko A., Gao F., Gillingham T.Z., Mathys H., Seo J., Kritskiy O., Abdurrob F. (2016). Gamma frequency entrainment attenuates amyloid load and modifies microglia. Nature.

[34] Sanchez-Mejias E., Nuñez-Diaz C., Sanchez-Varo R., Gomez-Arboledas A., Garcia-Leon J.A., Fernandez-Valenzuela J.J., Mejias-Ortega M., Trujillo-Estrada L., Baglietto-Vargas D., Moreno-Gonzalez I. (2020). Distinct disease-sensitive GABAergic neurons in the perirhinal cortex of Alzheimer’s mice and patients. Brain Pathol..

[35] Saiz-Sanchez D., De la Rosa-Prieto C., Ubeda-Banon I., Martinez-Marcos A. (2015). Interneurons, tau and amyloid-β in the piriform cortex in Alzheimer’s disease. Brain Struct. Funct..

[36] Ruden J.B., Dugan L.L., Konradi C. (2021). Parvalbumin interneuron vulnerability and brain disorders. Neuropsychopharmacology.

[37] Zhang X., Wu M., Cheng L., Cao W., Liu Z., Yang S.-B., Kim M.-S. (2025). Fast-spiking parvalbumin-positive interneurons: New perspectives of treatment and future challenges in dementia. Mol. Psychiatry.

[38] Tweedy C., Kindred N., Curry J., Williams C., Taylor J.P., Atkinson P., Randall F., Erskine D., Morris C.M., Reeve A.K. (2021). Hippocampal network hyperexcitability in young transgenic mice expressing human mutant alpha-synuclein. Neurobiol. Dis..

[39] Shu S., Xu S.Y., Ye L., Liu Y., Cao X., Jia J.Q., Bian H.J., Liu Y., Zhu X.L., Xu Y. (2023). Prefrontal parvalbumin interneurons deficits mediate early emotional dysfunction in Alzheimer’s disease. Neuropsychopharmacology.

[40] Hijazi S., Smit A.B., van Kesteren R.E. (2023). Fast-spiking parvalbumin-positive interneurons in brain physiology and Alzheimer’s disease. Mol. Psychiatry.

[41] Hijazi S., Heistek T.S., Scheltens P., Neumann U., Shimshek D.R., Mansvelder H.D., Smit A.B., van Kesteren R.E. (2020). Early restoration of parvalbumin interneuron activity prevents memory loss and network hyperexcitability in a mouse model of Alzheimer’s disease. Mol. Psychiatry.

[42] Wu Z., Wang Y., Chen W.W., Sun H., Chen X., Li X., Wang Z., Liang W., Wang S.Y., Luan X. (2025). Peripheral nervous system microglia-like cells regulate neuronal soma size throughout evolution. Cell.

[43] Henstridge C.M., Tzioras M., Paolicelli R.C. (2019). Glial Contribution to Excitatory and Inhibitory Synapse Loss in Neurodegeneration. Front. Cell Neurosci..

[44] Tsujioka H., Yamashita T. (2021). Neural circuit repair after central nervous system injury. Int. Immunol..

